# Improved Action Recognition with Separable Spatio-Temporal Attention Using Alternative Skeletal and Video Pre-Processing

**DOI:** 10.3390/s21031005

**Published:** 2021-02-02

**Authors:** Pau Climent-Pérez, Francisco Florez-Revuelta

**Affiliations:** Department of Computing Technology, University of Alicante, P.O. Box 99, E-03080 Alicante, Spain; florez@dtic.ua.es

**Keywords:** active and assisted living, action recognition, computer vision, spatio-temporal attention, deep learning, inflated convolutional neural networks

## Abstract

The potential benefits of recognising activities of daily living from video for active and assisted living have yet to be fully untapped. These technologies can be used for behaviour understanding, and lifelogging for caregivers and end users alike. The recent publication of realistic datasets for this purpose, such as the Toyota Smarthomes dataset, calls for pushing forward the efforts to improve action recognition. Using the separable spatio-temporal attention network proposed in the literature, this paper introduces a view-invariant normalisation of skeletal pose data and full activity crops for RGB data, which improve the baseline results by 9.5% (on the cross-subject experiments), outperforming state-of-the-art techniques in this field when using the original unmodified skeletal data in dataset. Our code and data are available online.

## 1. Introduction

Societies of countries in the organisation for economic co-operation and development (OECD) are faced with the challenge of increasing older population [1] as reported by multiple agencies [2,3,4]. This increase brings associated fears: how to keep welfare and provide care and health services for such a large population of older people, with ever-shrinking workforce.

Active and assisted living (AAL) technologies aim at ameliorating the situation by providing tools to older people, their caregivers, and health practitioners with the goal of supporting end users to stay independent for longer using information and communication technologies (ICTs). The European Union and other governmental bodies have recognised the importance of this field by funding specific calls for research into the development of related technologies, as noted by Calvaresi et al. [5].

## 2. Motivation

Action recognition, and more specifically the recognition of activities of daily living (ADLs) in the context of AAL, is a research field with much potential in terms of applications that could benefit older and dependent people: from creating a log gathering all activities that occur during the day for long-term behaviour analysis; or for inspection by caregivers; or for self-reflection by end users, remembrance, and therapy adherence (“did I take that pill today?”, “when did this happen?”); to assistance robots and cue-based systems that intervene when the user hesitates about the next step during the activity, or when a potentially dangerous activity is detected (not just falling, but intake of pills outside prescribed hours, leaving appliances running and forgetting about them, etc.).

However, to this day, and despite advances in the last few decades, it is still an ongoing effort in research to achieve activity recognition under realistic conditions, not only because more work needs to be carried out on the design and application of machine learning methods but also due to the lack of large, unconstrained, realistic datasets. Initially, datasets were very ‘staged’: actions were recorded showing the side of the body undergoing the greatest variation (mostly from the side, some frontal actions, such as ‘skip’), such as in the Weizmann dataset [6] or the KTH dataset [7]. Furthermore, these datasets had few action classes, and these were very distinctive from one another, and were performed mostly on a plain background. More recently, with the emergence of RGB-D sensors (Microsoft Kinect, Asus Xtion, PrimeSense, Orbbec Astra, etc.), several new datasets have appeared. The reader is referred to Firman [8], who has collected and categorised most of them. These are, in some cases, more specific to either gaming and sports, such as the Microsoft Research (MSR) Action 3D dataset [9] or the UTD-MHAD dataset [10]; or also daily activities recognition with the MSR Daily Activity dataset [11] or RGBD-HuDaAct [12]. However, most of these datasets are limited for data-driven learning, i.e., the most common approach lately, with the use of ‘deep learning’-based techniques requiring greater amounts of data. A proof of this is the fact that, for most datasets listed so far, researchers have had to use evaluation protocols involving leave-one-out cross-validation techniques. Larger efforts also exist, such as the Cornell activity datasets (CAD-60 and CAD-120) [13], or the Nortwestern-UCLA (NUCLA) dataset [14]. The largest of such datasets captured from multiple RGB-D sensors is the NTU dataset by Shahroudy et al. [15], as well as its later extension [16]. However, one could argue that these datasets, although evolved if compared to earlier datasets, are still very ‘unnatural’ or ’acted’, as they still have sets of repetitions of several action classes performed at set angles and captured in laboratory conditions mostly by young and healthy adults.

For AAL-related tasks, such as ADL recognition, general datasets for action recognition have too much inter-class variation, i.e., larger class separation, (i.e., recorded activities differ largely from one another), while having activities usually performed very similarly in terms of variation among actors (low intra-class variation). This is just the opposite of what is needed for recognition of ADLs, since there can be very similar classes that need to be distinguished correctly (e.g., eating, taking pill, drinking water), whereas end-users will not ‘perform’ homogeneously, but rather each will have very different ways of going about their daily routines.

For this reason, Das et al. [17] presented a dataset for action recognition with the particularities of ADLs: namely, one that has lower inter-class variation than usual in other general action recognition datasets, while having greater intra-class variation by different users. Their dataset consists of 16,115 videos, spanning 31 activity classes taken from 7 different views (not necessarily concurrently, though). More interestingly, their dataset is captured without giving the actors any cues about the actions to perform, since it is captured from ‘residents’ of a smart home setup. Furthermore, they do an initial proposal as to how to perform action recognition using this dataset as their benchmark. Given the complexity of the low inter-class variability, they propose a neural network architecture that incorporates the concept of ‘attention’ to focus on the finer details of the action (e.g., objects manipulated near the hands) in the spatial, as well as the temporal domains (e.g. bits of the video clips which are more relevant to determine the action class). They coin this approach as separable spatio-temporal attention network (separable STA). The architecture consists of two branches: one processing skeletal data using a three-layer LSTM (long short-term memory blocks)); and another taking spatio-temporal (XYT) volumes of RGB information, consisting of an I3D network (inflated 3D convolutional neural network, [18]). The LSTM branch is then attached to the spatio-temporal attention module, which learns spatial and temporal weights that are used, separately to modulate (each) the output of the layer before the global average pooling (GAP) of the I3D model. These modulated feature maps are then concatenated and passed through a 1×1×1 convolution and *softmaxed* to get the final one-hot output.

Skeletal data can be extracted from diverse sources. Initially, along with the arrival of RGB-D sensors, the first pose estimation algorithms from depth images were released: these were mostly based on the use of synthetic depth images to achieve real-time pose estimation from single depth images [19]. It was then possible to have quite accurate results with either 15 or 20 different joints, depending on the system (namely, Microsoft’s or OpenNI using Nite). More recently, with the advent of neural networks for pose estimation [20,21,22], it has been possible to obtain this information directly from RGB images, thus reducing the cost of the deployed sensors. An additional advantage is that with inference from neural networks it is possible to use images captured in full sun, as most RGB-D devices fail when used in presence of ‘interfering’ infrared sources. Either way, the skeletal data obtained often consists of N joints, and 3D data for each, yielding a 3×N vector encoding the skeleton. However, these points represent the person’s pose at the angle from the camera at which the activity was originally performed, thus creating extra variability between different samples of the same action class. One way of improving algorithm convergence during training is to reduce intra-class variability by means of data normalisation or simplification (dimensionality reduction). Simpler models might be employed, resulting in faster convergence, less resources needed, and faster inference during deployment. Skeletal data normalisation has been used in the past [15,23]. For instance, Chaaraoui et al. [23] propose a method to find the *Y*-axis rotation of the skeleton’s shoulders with respect to the sensor. This way, all skeletons will ‘face the camera’ after normalisation. Similarly, in the NTU dataset by Shahroudy et al. [15], pre-processing of all skeletons is performed to translate from camera coordinate system to the body coordinate system, with the origin of coordinates centred in the ‘spine’ joint. This process is then followed by a 3D rotation to fix the X axis parallel to the 3D vector from ‘right shoulder’ to ‘left shoulder’, and *Y* axis towards the 3D vector from ‘spine base’ to ‘spine’. The *Z* axis is then fixed as the new X×Y.

**Figure 1:**
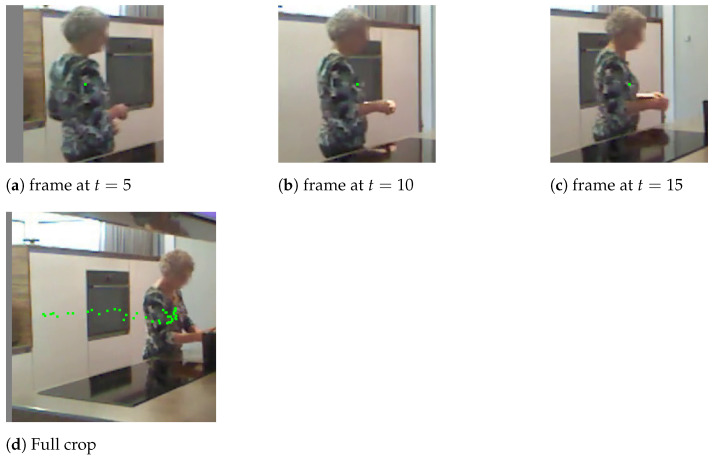
Demonstration of the ‘full crop’ concept. Top row (**a**–**c**) show the crops of three frames of a ‘Walk’ sequence 5 frames apart (green dots represent centre of detection). Bottom row (**d**) shows the full activity crop for the last frame of the same ‘Walk’ sequence (green dots represent centre of each detection throughout time).

There are two main limitations in the STA solution proposed by Das et al. [17]. The first one has to do with how skeletons are fed into the LSTM branch unchanged, i.e., without any rotation-based normalisation. This means there will be unnecessary intra-class variation due to the angle at which the skeletons have been extracted from the different capturing devices, making convergence of the networks harder. This paper proposes to apply a normalisation of the skeletons as a pre-processing step. The second one, has to do with how their I3D branch uses crops around each detection for each frame. In some cases, this limits the capability of the model to understand the action taking place, since the action is better represented by the displacement of the subject in space (e.g., in ‘Walk’ examples). This *spatial* displacement of the subject is better visualised by the network when focusing on the whole area. This paper introduces the idea of a full activity crop (“full crop”), taking into account the whole bounding box where the activity takes place. This can be better visualised in Figure 1: a woman is walking behind a kitchen counter (action label ‘Walk’). The legs are, therefore, occluded. Top row shows the crops around the detected subject at 5 frame intervals. A green point is used to show the centre of the detection bounding box. Note that there is no apparent movement of the subject (except the background changes). Bottom row shows the full activity crop for the last frame in the sequence. Green dots represent the centre of each detection throughout the activity. The trail on the bottom row image shows that displacement of the subject within the space is more prominent using this pre-processing for the the RGB XYT volumes.

The next section will further introduce the alternative pre-processing techniques that can be used to achieve better action recognition performance.

## 3. Alternative Skeletal and Video Pre-Processing

This section will introduce the main contribution of this paper, namely the alternative pre-processing techniques that can be used on the separable STA introduced by Das et al. [17], which can improve recognition of actions on the Toyota Smarthomes dataset. On the one hand, skeletal data, which is fed to the LSTM brach, will be normalised, rotating the skeletons so that they ‘look at’ the camera (rotation among the *Y* axis). There will also be a minor rotation on the *X* axis, to correct for the ‘tilt’ of the camera with respect to the ground. Additionally, crops around the detected person will be expanded to focus on the whole space where the activity takes place, i.e., the bounding area of the image comprising all the detections of the subject.

### 3.1. Skeletal Pre-Processing

In the Toyota Smarthomes dataset [17], skeletal data is not obtained from an RGB-D sensor, as done in other activity recognition datasets, such as the NTU dataset [15,16]. Rather, an LCR-Net (Localization-Classification-Regression) neural network [22] is applied to the RGB video clips for each action, thus obtaining skeletons for each frame. These skeletons show the rotation of the body as it appears in the image. The skeletal data is provided both in image pixel coordinates, as well as estimated 3D distances of each joint to the ‘spine’ centre. Depth information (*Z* axis) is, therefore, relative to this ‘spine’ joint, which is sitting on the origin of coordinates (0,0,0).

**Figure 2:**
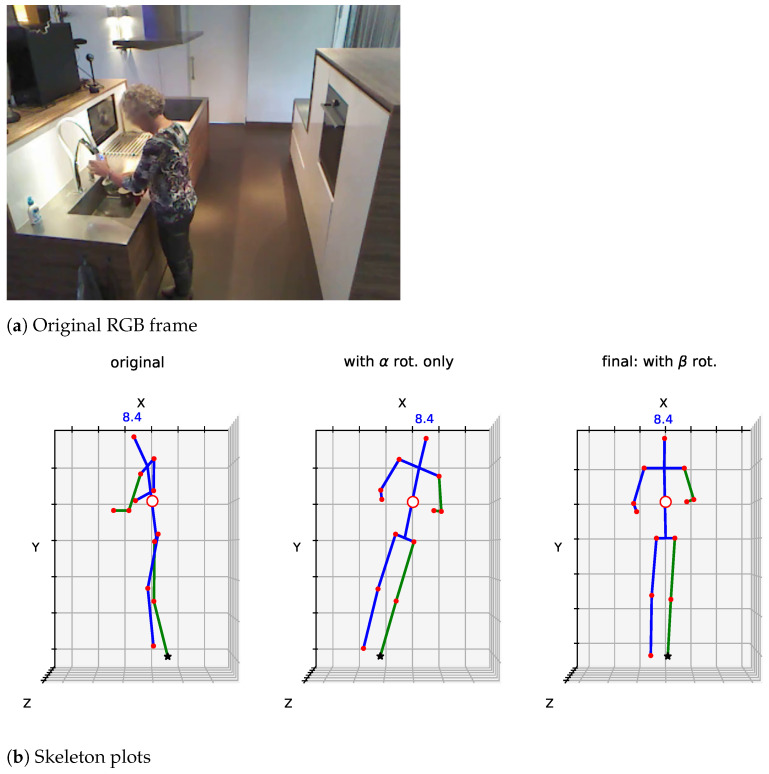
Proposed skeleton rotation at different stages. (**a**) shows the original video frame; (**b**) left: original skeleton as detected by LCR-Net, centre: skeleton rotated about the *Y* axis (intermediate step), right: skeleton fully rotated also about the *Z* axis.

The skeletal data provided has, therefore, one main disadvantage: the same action, performed from different camera views will look completely different, i.e., the body might be rotated in a particular way due to the distribution of furniture or appliances necessary to develop the activity in the home (e.g., ‘washing dishes’ will occur in front of the sink). This makes the approach too specific to the ‘current’ scenario, rather than pre-processing the skeletons so that regardless of camera view, the activity is ‘seen’ from a view-neutral standpoint. Furthermore, the detections of LCR-Net do not seem to correct for the angle of the camera with respect to the ground; therefore, as shown below (Figure 2), one part of the body (left or right, again depending on camera view) will be higher up (*Y* axis) than the other side. This might make the skeletons too specific for a camera view and could potentially reveal this information to the network during training.

With these limitations in mind, two rotations are then applied to the skeletons. The first one, to rotate all skeletons so that they are introduced to the network ‘facing forward’, i.e., rotating them about the *Y* axis, using an angle α, calculated from three skeleton joints $\vec{sl},\vec{sr},\mathrm{and}\;\vec{hr}$, which are the left and right shoulder and the right hip, respectively. These three joints are considered to conform the plane of the torso, and used to estimate its rotation with respect to the XY plane of the axes. The average ‘depth’ (*z* subindices) and average *x* values of joints to the left and right are used to calculate α:

(1) $$\alpha =\arctan\left(\frac{sl_{z}-\frac{\left(sr_{z}+hr_{z}\right)}{2}}{sl_{x}-\frac{\left(sr_{x}+hr_{x}\right)}{2}}\right).$$

The idea behind this rotation is that it will create a camera-independent view for all skeletons, therefore normalising them with regards of the view from which the skeleton was curated. Furthermore, it is worth noting that angle α is only calculated once at the beginning (at $t_{0}$), so that body rotations occurring naturally as part of the activity are not ‘over-corrected’.

Then, a second rotation is applied to compensate the angle of the camera with respect to the ground, which is tilted downwards in most cases in the dataset employed. However, because of the time-independent nature of LCR-Net detections, this angle changes slightly from frame to frame; therefore, this rotation β is calculated at each time frame (*t*), as:

(2) $$\beta_{t}=\frac{\beta_{s,t}+\beta_{h,t}}{2},$$

where $\beta_{s,\cdot }$ and $\beta_{h,\cdot }$ are two independent estimations for the angle between the shoulders ($\vec{sl}$, $\vec{sr}$) and hips ($\vec{hl}$, $\vec{hr}$), respectively:

(3) $$\beta_{s}=\arctan\left(\frac{sl_{y}-sr_{y}}{sl_{x}-sr_{x}}\right)\;\;\mathrm{and}\;\;\beta_{h}=\arctan\left(\frac{hl_{y}-hr_{y}}{hl_{x}-hr_{x}}\right).$$

Figure 2 shows the 3D skeleton obtained for a sample of a person as seen from the side (bottom row, left), estimated via LCR-Net from the RGB image (top row). The skeleton is then rotated about the *Y* axis (using the pre-calculated α angle); and also about the *Z* axis (on the XY plane), using $\beta_{t}$.

### 3.2. Video Data Pre-Processing

The original paper of Das et al. [17] does video clip pre-processing by cropping the area around the detection of single shot multi-box detector (SSD) network model [24]. However, this has two disadvantages: first, this is a box detector, rather than a mask detector; therefore, crops might not be as accurate; and second, this is not a “historical” cropping, i.e., taking into consideration dynamics of the detected person throughout time. In this paper, two alternatives to this are presented. One is using Mask-RCNN (mask region-based convolutional neural network) [25], as an alternative to SSD. The other is to do a “full crop” of the part of the image where the human action to be recognised happened. That is, integrating the whole space where all human detections have appeared in the image throughout history (time). The resulting bounding box for the action is defined by the top-left ($p_{TL}$) and bottom-right ($p_{BR}$) corners (points), which are the minimum x,y, and maximum x,y coordinates of all detections through time (*t*), respectively. That is:

(4) $$\vec{p_{TL}}=\min_{i=1..t}\left(x_{i},y_{i}\right);\;\vec{p_{BR}}=\max_{i=1..t}\left(x_{i},y_{i}\right).$$

**Figure 3:**
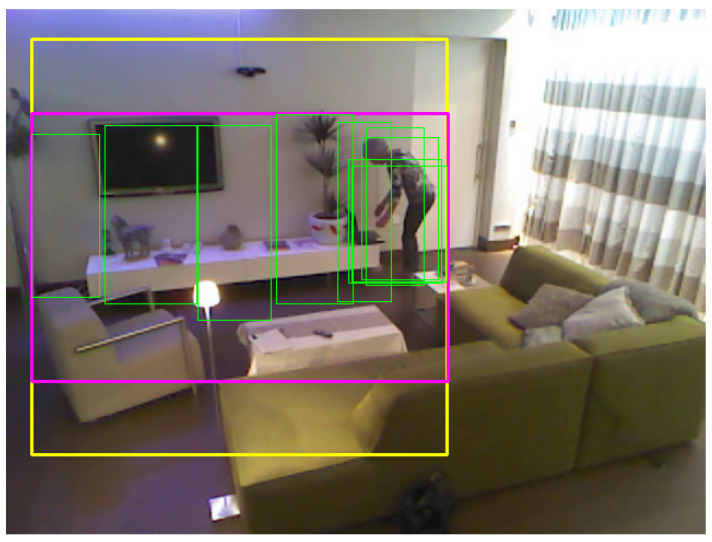
Frame from a ‘Walk’ sequence showing the *full crop* concept: a crop involving the full area where the activity takes place (bounding box in purple). The yellow bounding box shows the square area of the final image crop. Bounding boxes of each separate detection shown in green. For visibility, only one in every 10 detections (green bounding boxes) is shown.

This bounding box is shown in purple in Figure 3. Because this bounding box is not necessarily square, but the I3D network expects a square image as input, a square crop enclosing the purple bounding box is then used to crop the image (shown in yellow in Figure 3). To calculate it, the centre of the purple bounding box is found, and the larger side of it is used as the size of the side of the enclosing square bounding box. When the resulting bounding box falls partially outside the image canvas, padding is added containing grey pixels (RGB values of 128).

As opposed to this full activity crop, in the other results obtained, the protocol is to calculate the square crop around each detection bounding box (shown in green in Figure 3) separately.

### 3.3. Experimental Setup

The LSTM branch is trained using Adam optimiser. The implementation used is that of Das et al. [26,27] which is initialised with a learning rate (LR) of 0.005 ($5\times 10^{-3}$). However, in the experiments in this paper (see the ‘Supplementary Materials’ section at the end for available code and data) the LR is reduced by two orders of magnitude to $5\times 10^{-5}$. This is because class weighing is introduced for training, since the training set is heavily unbalanced. Training is left for 300 epochs, and the best result for the validation set then used to run on the test set. Dropout and other parameters are left unchanged.

Learning rate adjustments have also been used in the I3D branch implementation [28], which uses stochastic gradient descent (SGD). Again, the original LR was set to $10^{-2}$ and reduced by two orders of magnitude to $10^{-4}$. Training runs for 50 epochs, proceeding similarly as above.

Regarding the separable STA, it has been re-implemented following instructions in their paper [17]. Again the initial LR for Adam is reduced by two orders of magnitude from the original value of 0.001 ($=10^{-3}$) to $10^{-5}$. Since the protocol of Das et al. [17] establishes that the separable STA layers are trained *jointly* with the I3D, the layers of the latter are left as trainable. This is labelled in the results tables as ’jointly’. In a previous work by Das et al. [29], the authors intialised the STA component with equal values for the attention weights, and Gaussian for the rest of the layers. Furthermore, they performed a two-stage training consisting of $N_{1}$ epochs with trainable branches and $N_{2}$ epochs with ‘frozen’ weights on the branches. Neither of these is done here, since their paper [17] does not mention them.

## 4. Results and Discussion

This section will introduce the results for the cross-subject (CS) and cross-view (CV${}_{2}$) experiments. Additionally, a comparison with other state-of-the-art techniques is also presented.

### 4.1. Cross-Subject Evaluation

Table 1 shows the results for the cross-subject experiment. Train, validation, and test subsets follow the same protocol as Das et al. [17]. Mean per-class accuracies (MPCA) and classification accuracies are provided. ‘Baseline’ refers to the result of re-running the original experiments, or in the case of I3D, using crops from Mask-RCNN (since the SSD crops were not provided originally with the dataset). Each component (branch) of the network has been trained separately for classification and then used (frozen) in the separable STA.

Regarding the LSTM results, it is worth noting that the original paper presenting the dataset [17] does not provide MPCA results of the implemented 3-layer LSTM architecture, but rather that of Mahasseni and Todorovic [30]. The baseline result is lower than the provided result (30.7% vs. 42.5%), but the rotation applied improves results (34.5%). Additionally, given the results, another experiment adding hand-to-hand and hand-to-head distances (3 values in total) to the skeletal data feature vector further improve the results (labelled as ‘Rot. + Ds’: 36.7%). This shows that further feature engineering might be beneficial in some cases.

Regarding classification accuracies, it is worth mentioning that the introduction of class weighing during training might reduce the accuracy while keeping the same MPCA, showing how very unbalanced results favouring more common classes results in much higher accuracies that artificially inflate the results. This is shown in an extra experiment (row 3 in Table 1), in which class weighing is removed, obtaining 59% (vs. 54.5%) accuracy.

When looking at the results for the I3D branch in isolation, it can be observed that the re-run of the original experiment with Mask-RCNN crops yields better results (58.4% vs. 53.4%). This can be attributed to the more accurate bounding boxes of the method (i.e., ‘tighter fit’), or the fact that Mask-RCNN can detect partially occluded people better than the SSD used by Das et al. [17], therefore having more frames with detected subjects than in their work. When adding the full activity crop pre-processing (‘Full crop’ on the table), results further improve to 63.4%, thus being even better than the result reported on their paper for the separable STA (54.2%). It needs be said that the improvement of separable STA with respect to the I3D branch in their paper is only 0.8%, i.e., the attention mechanism does not seem to provide much improvement in terms of MPCA.

**Figure 4:**
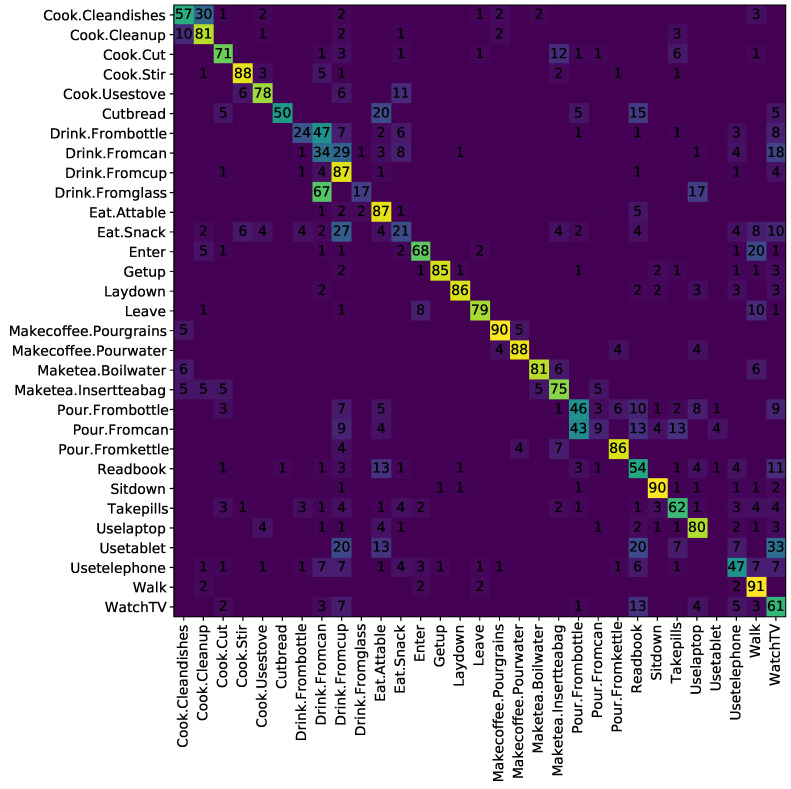
Confusion matrix (in %) for the best cross-subject separable STA result (‘Rotation’).

Finally, when taking the pre-trained branches, and feeding their outputs into the separable STA, results improve with respect to the reported values by 0.2% (54.6%) when using a ‘Baseline’ approach (note: using Mask-RCNN crops instead of SSD); or further to 62.8% when using rotated skeletons and the distances described above (‘Rot. + Ds’); and even further when using just rotation to 63.7% or 63.5% when using ‘Both’ rotation and full activity crops. Similarly to the results reported by Das et al. [17], improvement over the I3D branch is marginal (0.1–0.3%), which seems to indicate that the attention network is not contributing much to improve the end result. Please also note that, all MPCA scores for the separable STA are higher than those reported by Das et al. [17], regardless of the overall recognition accuracy, meaning the presented results are better for a variety of classes (e.g., ‘Drink from cup’, ‘Eat at table’, or those between ‘Enter’ and ’Make tea, insert tea bag’), not just over-represented ones (e.g., ‘Walk’). There is also less confusion around cooking-related activities (e.g., ‘Cook.Stir’) (Figure 4).

### 4.2. Cross-View

Table 2 shows the results for the cross-view (CV${}_{2}$) experiment. Again, split of sequences into train, validation, and test follow the protocol of Das et al. [17]. As in the CS experiment, ‘Baseline’ represents the re-run of the experiments, but using Mask-RCNN crops for the I3D branch.

Starting with the LSTM results, it can be seen that, for this experiment in particular, skeleton rotation proves very useful, as results almost double from 17.2% to 30.1%. This can be explained by the fact that rotating skeletons so that they are viewed from a camera-neutral standpoint clearly benefits the training process by reducing the intra-class variations that occur when trying to learn the same activity class as seen from different views.

With regards to I3D, however, in this case, the ‘Baseline’ re-run of the experiment provides a lower score (40.0%). The full activity crop, however, improves results to 48.2%, which is 3.1% above the result of 45.1% reported by Das et al. [17].

**Figure 5:**
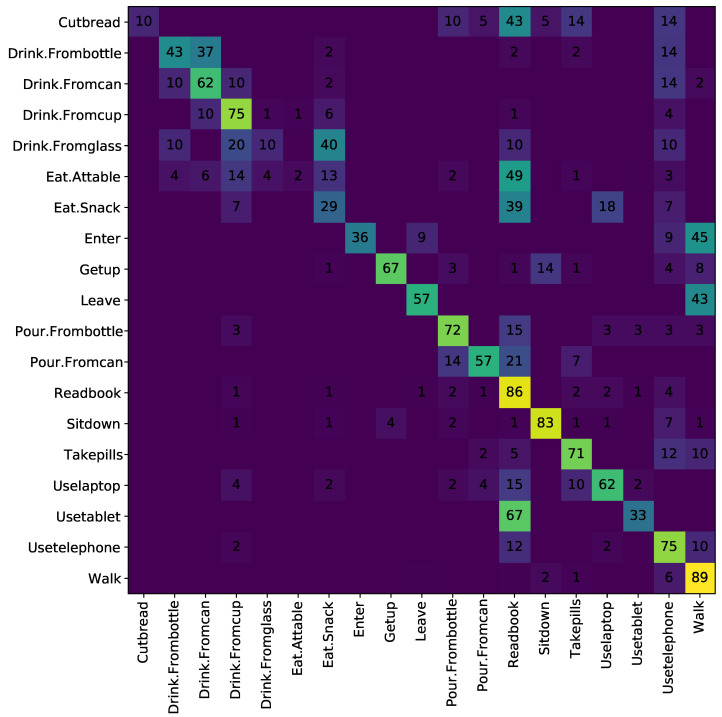
Confusion matrix (in %) for the best cross-view separable STA result (‘Both, jointly’).

For the separable STA joint network, as in the CS experiment, the results mostly replicate those of the I3D branch. For instance, the ‘Rotation’ result is almost the same (40.9%). This is preoccupying, since it leads to believe that the attention network is not leading to improvement. When using the full crop variant of I3D (48.2%), it then increases to 50.3%, which is comparable to the results by Das et al. [17]. Finally, if the I3D branch is left as trainable (*thawed*), results further improve to 53.6% (3.3% improvement). Figure 5 shows the confusion matrix for this case, with improvements for some classes with respect to results reported by Das et al. [17], e.g., ‘Get up’, ’Leave’.

### 4.3. Comparison to Other Methods

Finally, Table 3 shows the results when comparing the proposed pre-processing techniques on separable STA to other methods in the literature.

The Video-Pose Network (VPN) model of Das et al. [31] focuses on a shortfall of separable STA, which is that the pose and RGB information are not coupled: The LSTM branch is used to assist the attention block, to modulate the I3D output spatially and temporally, and these two modulated outputs are then concatenated and fed to the final classifier. However, there is no embedding (i.e., spatial correspondence, or coupling) between pose data, and RGB data. The VPN network focuses on this need, and consists of two main elements: an spatial embedding module to calculate joint to image-region correspondences, and an attention network of similar nature than that used in separable STA [17]. The results show that VPN outperforms separable STA (60.8% vs. 54.2% on CS; and 53.5% vs. 50.3% on CV${}_{2}$). Nonetheless, keeping the separable STA architecture, using proposed pre-processing methods, results improve further to 63.7% (on CS, with rotation), or 53.6% for CV${}_{2}$ using both.

Ryoo et al. [32] present AssembleNet++, an improvement on AssembleNet [35] that uses a multi-stream architecture with self- and peer- attention mechanisms. Their model uses three different modalities as inputs, namely: RGB data and optical flow, as well as object segmentation model trained pre-trained with the ADE-20K dataset. Their model is otherwise trained from scratch and achieves an overall classification accuracy of 80.6%, with a MPCA of 63.6%. This demonstrates that object segmentation, i.e., information about objects present in the scene helps improve recognition of activities, specially when those are very similar (e.g., ‘drink from cup’ v. ‘drink from glass’). Regardless, the proposed pre-processing techniques suffice on the separable STA model to achieve comparable results: 63.5% when using rotation of skeletons and full activity crops; or 63.7% when using rotation of skeletons only.

Finally, a very recent paper by Yang et al. [34] proposes a pose aggregation and refinement system, consisting on the use of several pose estimation algorithms (LCR-Net++ [22], AlphaPose [36], and OpenPose [37]), and a selective spatio-temporal aggregation (SSTA) mechanism that will select the best combination of skeletal data available. The resulting skeletons are more stable along time and regressed from a series of pre-clustered ‘anchor’ poses (similar to ‘key poses’ of Chaaraoui et al. [38]). With these refined poses, a weakly-supervised algorithm (pose refinement system, or PRS) is used to improve the results of LCR-Net++, so that not all pose estimators have to be used at every frame. As a consequence, the skeletons employed after applying PRS are a different set of skeletons to that used in this paper, and the others reviewed so far. This makes comparison of results difficult, since it would be necessary to re-run all other algorithms with this new set of skeletons for fair comparison (for this reason, the results using PRS appear greyed out in Table 3).

Two different approaches are tried by Yang et al. [34]: one using only pose-based information and using Adaptive Graph Convolutional Networks (AGCNs). They compare two-stream AGCN (2s-AGCN, from Shi et al. [33]) with and without PRS-improved skeletons, as well as expanding it to 5 channels (‘5C-AGCN’ results), which further improves their results on pose data only; the other, uses the VPN network proposed by Das et al. [31], adding PRS skeletal data. The latter achieves an MPCA of 65.2% for CS, and 54.1% for CV${}_{2}$. Nonetheless, the proposed pre-processing mechanisms still show the second best results, when compared to VPN with PRS (only 1.5%, and 0.5% below, respectively), at 63.7%, and 53.6%.

## 5. Conclusions

When looking at non-PRS results, the proposed methodology results in improved results for the Toyota Smarthome dataset, using the model proposed along with its publication, i.e., separable STA. This is better than other models that do not use pose information, but use seemingly very informative data, such as object segmentations, as done in AssembleNet++ [32]. Moreover, results are better for CS and comparative for CV${}_{2}$ when compared to VPN [31], which uses pose-to-image to couple both modalities. Improvement over the baseline separable STA [17] is of 9.5% for CS, and 3.3% for CV${}_{2}$.

Future work involves using PRS-enhanced skeletal data, to assess the improvement provided by the proposed method. Given that, particularly for CV${}_{2}$, pose-based recognition is improved almost two-fold (×1.75). Furthermore, it is very likely that the pre-processing techniques employed in this paper would benefit the results reported by Yang et al. [34] even further.

Additionally, and regarding privacy-aware algorithms for action recognition, it would be interesting as future work to replace all humans in RGB images by body-part labelled avatars (e.g., using DensePose [20]), thus simplifying (i.e., reducing intra-class variation) the particularities of each individual. This could be done by impainting the person in the RGB image, and using a multi-stream network with the dense poses as a separate stream, or directly by ‘drawing’ them on the RGB space. Studying how a dense pose (i.e., mesh-like structure) compares to a *sparse* one (joint-only pose) for action recognition is also interesting in that regard. This might lead to privacy-preserving AAL applications that improve end user acceptance of these technologies in ageing societies needing them the most.

## Figures and Tables

**Table 1:** Results for the cross-subject evaluation (CS). ‘Both’ refers to rotation with no distances and full crop (jointly-trained, i.e., leaving I3D branch layers as trainable). Results provided in mean per-class accuracy (MPCA) and overall classification accuracy. Best result in bold.

Component	Variant	MPCA (in %)	Accuracy (%)
LSTM	Das et al. [17]	42.5 [30]	—
	Baseline	30.7	53.4
	Rotation	34.5	**54.5** (59.0) *
	Rot. + Ds	**36.7**	54.1
I3D	Das et al. [17]	53.4	—
	Baseline	58.4	73.0
	Full crop	**63.4**	**74.3**
Separable STA	Das et al. [17]	54.2	75.3
	Baseline	54.6	71.1
	Rot. + Ds	62.8	74.0
	Rotation	**63.7**	76.5
	Both, jointly	63.5	**77.1**

* without class-weighing.

**Table 2:** Results for the cross-view evaluation protocol (CV${}_{2}$). ‘Both’ refers to rotation with no distances and full crop, either ‘jointly’-trained (i.e. I3D layers as trainable) or with ‘frozen’ branches. Results in mean per-class accuracy (MPCA) and overall classification accuracy. Best result in bold.

Component	Variant	MPCA (in %)	Accuracy (%)
LSTM	Das et al. [17]	17.2 [30]	—
	Rotation	**30.1**	**46.3**
I3D	Das et al. [17]	45.1	—
	Baseline	40.0	53.4
	Full crop	**48.2**	**63.1**
Separable STA	Das et al. [17]	50.3	68.2
	Rotation	40.9	53.0
	Both, frozen	50.3	**65.7**
	Both, jointly	**53.6**	65.6

**Table 3:** Comparison to state-of-the-art methods. Mean per-class accuracy (in %).

Method	CS	CV${}_{1}$	CV${}_{2}$
separable STA [17]	54.2	35.2	50.3
VPN [31]	60.8	43.8	53.5
AssembleNet++ [32]	63.6	—	—
2s-AGCN [33,34]	57.1	22.1	49.7
2s-AGCN+PRS [34]	60.9	22.5	53.5
5C-AGCN+PRS [34]	62.1	22.8	54.0
VPN+PRS [34]	65.2	—	54.1
Proposed (best values)	63.7	—	53.6

## Data Availability

Restrictions apply to the availability of these data. Data was obtained from Srijan Das (INRIA, France) and are available at https://project.inria.fr/toyotasmarthome/ with their permission. The processed data presented in this study are available at https://github.com/DAIGroup/improved_HAR_on_Toyota.

## Supplementary Materials

The code, data, and results (along with additional confusion matrices) are available online at https://github.com/DAIGroup/improved_HAR_on_Toyota.
